# Multiobjective distribution system operation with demand response to optimize solar hosting capacity, voltage deviation index and network loss

**DOI:** 10.1038/s41598-024-82379-7

**Published:** 2025-01-02

**Authors:** Kabulo Loji, Sachin Sharma, Gulshan Sharma, Tanuj Rawat

**Affiliations:** 1 Department of Electrical Power Engineering, Durban University of Technology, Durban, 4001 South Africa; 2 Department of Electrical and Electronics Engineering, Manipal Institute of Technology, Manipal Academy of Higher Education, Manipal, Karnataka India; 3 Department of Electrical Engineering Technology, University of Johannesburg, Johannesburg, 2006 South Africa; 4 GE Renewable Energy, Noida, India

**Keywords:** Multi-objective optimization, Crow search optimization algorithm, Solar photovoltaic, Demand response, Energy science and technology, Engineering

## Abstract

In this research, demand response impact on the hosting capacity of solar photovoltaic for distribution system is investigated. The suggested solution model is formulated and presented as a tri-objective optimization that consider maximization of solar PV hosting capacity (HC), minimization of network losses (Loss) and maintaining node voltage deviation (V_Dev_) within acceptable limits. These crucial objectives are optimized simultaneously as well as individually. To assess the efficacy of the solution, different multi-objective case studies are scrutinised based on the combinations of (i) HC and Loss, (ii) HC and V_Dev_, (iii) Loss and V_Dev_, (iv) HC Loss and V_Dev_ simultaneously with the effect of demand response. The multi-objective research problem is formulated as non-linear and non-convex programming approach. To solve this complex problem, the modified crow search optimization (MCSO) is proposed. The MCSO achieved the 0.0714 MW of network loss with the optimal integration of distributed generation and is comparable to the well-established optimization algorithms available in literature. From the simulation results, it is found that HC is 3322.31 kW, V_Dev_ is 0.4982 p.u and system losses is 1314.86 kWh with demand response program when all the objectives are simultaneously optimized. The simulation outcomes highlight the superiority of the MCSO over others. The application results show the benefits and the beauty of proposed research work.

## Introduction

### Motivation and background

Energy consumption is a significant factor in the climate change brought on by greenhouse gas emissions, which has drawn attention from all around the world. As a result, numerous nations have committed to meeting carbon neutrality targets before 2050. Moreover, reaching carbon neutrality and total energy consumption goals can be greatly impacted by increased penetration of renewable energy sources for power generation and consumers awareness in the demand response programs. Due to intensive research and development efforts, the exploitation of renewable energy technologies, including solar photovoltaic (PV) generation, wind power generation, hydropower, and biomass energy generation, has seen significant improvement. Solar energy has evolved significantly with the introduction of high-efficiency photovoltaic cells and sophisticated solar concentrators. PV panels are a promising technology, contributing 12% of the world energy production in 2023. However, some flexible technology such as energy storage devices are required due to the intermittent nature of PV power and the mismatch between load profiles, which adds to the overall expenses of the renewable energy system. The demand response are flexible operating strategies that have the capacity to absorb the uncertainty of solar power with minimal cost as comparison to battery energy storage system ^3^. Renewable energy technologies, energy storage, and flexible scheduling of node wise consumer demand can greatly improve the objectives of distribution system operators.

### Literature review

The last decade, has seen a significant increase in renewable energy-based distributed generators (DGs) within power distribution grids ^1^. Shifting from conventional generation to DGs brings notable benefits, including enhanced voltage profiles, reduced losses, and lower capital costs. While some of this new generation capacity is provided by utility-scale PV and wind turbines, the majority is expected to come from residential rooftop PVs, which are much closer to the load centres ^2^. However, traditional distribution systems were not designed to handle widespread PV installations. This transition poses considerable operational challenges, as high levels of DG penetration can cause issues such as undesirable voltage flicker and accelerated equipment aging due to excessive voltage regulation activities, to name a few. Utilities urgently need an efficient strategy for widespread PV deployment that avoids planning violations. To tackle these issues, it is vital for utilities to accurately assess the system hosting capacity (HC) to understand the impact of increased DG penetration, plan for their efficient deployment, and carry out all the necessary upgrades to ensure reliable system operation ^3–5^. HC refers to the maximum amount of power generation that a system can support without exceeding any operational standards ^6^. In line with this definition, the Electric Power Research Institute (EPRI) recommends simulation-based methods that model each feeder and utilize screening tools to assess power quality and reliability issues, thereby determining HC at different locations ^7^. The simulation incrementally raises the PV penetration level during power flow analysis until one of the constraints is breached. The maximum level of PV generation before this breach is identified as the hosting capacity ^8–10^. This concept has already been implemented in some commercial software for utilities such as CYME, that conducts the per-node HC analysis by; (1) selecting a numerical iteration method for power flow, (2) choosing the relevant operational constraints, and (3) performing extensive simulations with increasing local PVs to determine HC. However, focusing solely on one bus neglects the interdependencies within the system, which are crucial for an accurate HC assessment and determination. Additionally, it is numerically impractical to exhaust all possible scenarios through simulations alone. From the foregoing, mathematical model-based methods are more and being employed for the evaluation of HC. In ^11^, the authors formulate the HC problem as an AC optimal power flow (ACOPF) problem, which was extended as a multi-period ACOPF by the works in ^12,13^. Given the complexity of solving ACOPF with numerous constraints, Ref. ^14,15^ propose using sensitivity analysis as an approximation to identify critical factors such as voltage magnitudes, voltage angle differences, network topology, load size, voltage variations, and generation locations ^16,17^. In ^18^, the authors suggested the use of a probabilistic model to account for these crucial factors ^18^. In light with these considerations, various additional strategies are proposed for HC enhancement, including on-load tap-changers (OLTC) ^19^, various reactive power control strategies ^20,21^, adoption of static and dynamic network re-configurations, integration of soft open points through power-electronic devices and most critical is the incorporation of real-time information ^22^. Since HC analysis occurs during the planning phase, effective control measures are typically assumed ^23–25^. Moreover, economic factors can be modelled to perform a cost–benefit analysis aimed at maximizing the HC for specifically solar PV ^26^. In ^27^, an efficient modified bald eagle search optimization algorithm is developed for the distribution system to minimize the power loss while maintaining the node voltage and line current limits. The jellyfish behaviour-based metaheuristic optimization algorithm is developed in ^28^ to minimize the power loss and improve the voltage stability index considering multiple DGs for distribution systems. The particle swarm optimization and genetic algorithm is proposed in ^29^ to minimize the active and reactive power loss with the consideration of seasonal load profile. In ^30^, authors proposed the multi-objective african vultures optimization algorithm considering hybrid distributed generation to optimize the system cost and reliability. The technique for order preference by similarity to the ideal solution technique is used to determine the one optimal solution from pareto optimal solutions. In ^31^, authors developed the multi objective problem considering wind, and photovoltaic generation to optimize the emission, cost of energy and penetration of renewable energy. The authors of ^32^ employed the improved binary African vultures optimization for the unit commitment problem. The uncertainty modelling of wind power using auto-regressive moving average model is addressed in ^33^ by developing the unit commitment problem. However, this uncertainty of renewable energy sources causes many issues for the operation of distribution system. Therefore, to minimize the impact of these uncertainty mostly previous research available in literature uses the battery energy storage system. But it increases the system investment cost. Moreover, the effective utilization of demand response (DR) strategies with these uncertain renewable energy sources reduces the sizes of battery energy storage system to some extent.

On the other side an improper use of DR can result in increased network losses and voltage imbalance, as mentioned in ^34^. While DR depends on consumer preferences, integrating it with advanced power control strategies can reduce energy loss due to curtailment. A coordinated approach that combines DR with an OLTC featuring independent phase tap control has proven to be effective for voltage management in a real-world three-phase four-wire suburban low-voltage network in Australia ^35^. This method increased a 40 kW PV HC to 65 kW, while concomitantly, achieved a voltage magnitude improvement, reduced unbalance, lowered compensation costs, and minimized comfort level violations during DR implementation. In ^36^, the authors examine the potential of DR in increasing the HC of solar PV system to find that even rudimentary DR control methods, although requiring detailed information on PV and load distribution, as well as PV penetration along the feeder length, can enhance the HC of PV. In the work scenario referred to above, DR implementation achieved to increase HC from 28.57 to 52.78% of the annual energy consumption, but only in particular when applied beyond 50% PV penetration. Hence, DR has the capability to unlock HC increase of solar PV but necessitate further research and investigations particularly with regards to the model and capacity of the distribution network. Furthermore, advance multi-objective optimization techniques will play a crucial role in considering various distribution parameters and constraints while increasing the HC of solar PV with active participation of DR program. The purpose of this article is to create a robust optimization model that present the best operational planning schedule and tracking operational status against consumers flexible demand while simultaneously determining the capacity of uncertain PV scenarios to achieve DS multi-objectives planning and operation of the DS.

### Contribution

The main contributions of this manuscript are as follows:

1. A novel multi-objective operational planning problem for the distribution system operator is developed that integrates uncertain solar PV generation together with the effect of flexible node wise consumers demand.
2. The performance of the distribution system is conducted to simultaneously optimize hosting capacity, voltage deviation index and network losses respectively.
3. To analyse the efficacy of the developed optimization model, seven different cases are framed on the basis of different combinations of the objectives. In case 1, the focus of the operation planning problem is to maximize PV HC. In case 2, the considered objective is to minimize the network loss and in case 3, to minimize the V_Dev_. Cases 4 to 7 are relevant to multi-objective considerations. Indeed, in case 4, the multi-objective problem is formulated to optimize the solar PV HC and the network losses for the distribution system. Case 5 optimizes PV HC and V_Dev_, while case 6 is dealing with the multi-objective formulation to determine an optimal solution focusing on network losses and V_Dev_. In case 7, the multi-objective framework in which solar PV HC, V_Dev_ and, network losses are simultaneously considered is suggested and investigated.
4. The MCSO is developed in this work to obtain the optimal scheduling of proposed energy system while considering imperative technical constraints such as DR, power balancing constraints, node voltage limit and HC of PV. The MCSO algorithm proficiency and robustness are assessed by comparing it to other widely used algorithms in literature.

The remaining section of the paper is organized as follows. “Problem formulation” provides the problem formulation. “Validation of MCSO” cover the MCSO for multi-objective operation problem. "Results discussion” includes a validation of MCSO. “Conclusion” presents the result section and Sect. 6 summarizes the conclusions.

## Problem formulation

Optimal hosting capacity of solar photovoltaic in an active DS is challenging for distribution system operator due to their unpredictable nature. The distribution system engineers aim to fulfil several goals with a single practical solution that benefits all the stakeholders both technically and financially. The proposed work creates a multi-objective function that includes daily vital objectives for an active distribution system. The objective function involves maximize the hosting capacity of solar photovoltaic (HC), minimize the network loss (P_L_) and node voltage deviation (V_Dev_).

### Maximize the hosting capacity of solar photovoltaic (HCSP)

As the globe moves toward sustainable energy options, incorporating solar PV reduces greenhouse gas emissions and reliance on fossil fuels. Furthermore, the decreasing cost of solar technology and the potential for localized energy generation empower customers while improving energy security. Power systems that promote increased optimal solar integration can improve the operation of distribution system. Therefore, maximizing the hosting capacity of solar is considered as one of the objectives and is framed as follows:

1 $$HC = \max \sum\limits_{{m \in \Psi^{PV} }} {C_{m}^{PV} }$$

### Minimize the network losses

The distribution system has a lower energy efficiency than the transmission system. This leads to a loss of annual utility revenue. So, minimizing energy losses in a distribution system is usually a top priority for distribution engineers. As a result, this is an objective function considered in the works that is modelled as ^37^:

2 $$P_{L} = \frac{1}{2}\sum\limits_{t = 1}^{24} {\left[ {\sum\limits_{m = 1}^{N} {\sum\limits_{n = 1}^{N} {\frac{1}{{X_{m,n} }}\left( {V_{m,t}^{2} + V_{n,t}^{2} - 2V_{m.t} V_{n,t} \cos (\delta_{n,t} - \delta_{m,t} )} \right)} } } \right]}$$

### Minimize the node voltage deviation

The power utilities are reluctant to allow the network to have a low node voltage profile since it may effect the linked devices. As a result, utility engineers strive to keep voltage profiles within defined boundaries. Thus, this objective function is considered in this study and drafted as ^37^.

3 $$\min V_{Dev} = \sum\limits_{{t = \Psi^{T} }} {\sum\limits_{{m \in \Psi^{N} }} {\left| {V_{m.t}^{2} - V_{setpt}^{2} } \right|} }$$

### Multi-objective for the distribution system operator

The overall multi-objective considered for the developed problem is the combination of network loss, node voltage deviation and PV hosting capacity and is framed as follow:

4 $$\min f = \omega_{1} P_{L} + \omega_{2} V_{Dev} - \omega_{3} H_{C}$$

### Constraints

The constraints considered for the developed problem are presented from Eqs. (5) to (14) and are defined as follows:

#### Power balance

The Eqs. (5) and (6) are represents the real and reactive node power balance and are defined as follows ^37^:

5 $$P_{m,t}^{GRID} + P_{m,t}^{PV} + P_{m,t}^{BESS,dsch} - P_{m,t}^{BESS,ch} - P_{m,t}^{load} = \sum\limits_{{m \in \Psi^{N} }} {\sum\limits_{{n \in \Psi^{N} }} {V_{m,t} Y_{mn,t} V_{n,t} } } \cos (\phi_{mn,t} + \delta_{n,t} - \delta_{m,t} ) \quad \forall m, t$$

6 $$Q_{m,t}^{GRID} - Q_{m,t}^{load} = - \sum\limits_{{m \in \Psi^{N} }} {\sum\limits_{{n \in \Psi^{N} }} {V_{m,t} Y_{mn,t} V_{n,t} } } \sin (\phi_{mn,t} + \delta_{m,t} - \delta_{n,t} ) \quad \forall m, t$$

#### Voltage limit

The maximum and minimum node voltage deviation constraint is represented in Eq. (7) and is defined as follows ^38^ :

7 $$V_{m}^{\min } \le V_{m,t} \le V_{m}^{Max} \quad \forall m, t$$

#### Hosting capacity

The Eqs. (8) and (9) are represents the constrains related to the hosting capacity of solar photovoltaic and are framed as follows:

8 $$0 \le P_{n,t}^{PV} \le C_{n}^{PV} P_{n,t}^{PV,p,u,} , n \in \Psi^{PV}$$

9 $$\sum\limits_{{m \in \Psi^{PV} }} {\sum\limits_{{t \in \Psi^{T} }} {P_{m,t}^{PV} \ge \sigma \sum\limits_{{m \in \Psi^{PV} }} {\sum\limits_{{t \in \Psi^{T} }} {C_{m}^{PV} P_{m,t}^{PV,p.u.} } } } }$$

#### Demand response

Equations (10)–(14) represent DR constraints. The Eqs. (10) and (11) show how to determine active and reactive demand after the implementation of DR depending on flexibility and demand. Constraints (12) and (13) ensure that the active and reactive energy levels stay constant before and after DR. Constraint (14) indicates boundaries on demand flexibility.

10 $$P_{n.t}^{load,aDR} = P_{n,t}^{load,bDR} \kappa_{n,t} \quad \forall n, t$$

11 $$Q_{n.t}^{load,aDR} = Q_{n.t}^{load,bDR} \kappa_{n,t} \quad \forall n, t$$

12 $$\sum\limits_{{t \in \Psi^{T} }} {P_{m,t}^{load,aDR} } = \sum\limits_{{t \in \Psi^{T} }} {P_{m,t}^{load,bDR} } \quad \forall n, t$$

13 $$\sum\limits_{{t \in \Psi^{T} }} {Q_{m,t}^{load,aDR} } = \sum\limits_{{t \in \Psi^{T} }} {Q_{m,t}^{load,bDR} } \quad \forall n, t$$

14 $$1 - \kappa_{m,t}^{Max} \le \kappa_{m,t} \le 1 + \kappa_{m,t}^{Max} \quad \forall m, t$$

##### MCSO for multi objective operational problem

The large-scale, real-world, mixed-integer, non-linear operational issues of increasing the hosting capacity of photovoltaic for distribution system under consideration is challenging. Therefore, it is difficult to solve the same problem effectively by using conventional optimization methods. The MCSO is a novel metaheuristic technique used in this work. The crow search optimization algorithm (CSO) was inspired by the crafty strategies used by crows. The crucial element utilized in this optimization process is the crow tendency to store extra food in specific places and be able to collect it when needed. When a crow follows another crow in quest of hidden food, two phases are developed based on the possibility of awareness. In the first stage, assume that the followed crow is unaware that it is being followed, that will enable the follower to discover the location of the food source and consequently, the quest will begin just in the neighbourhood. When however, a crow detects that it is being followed by a different crow or a group of crows, its second phase begins, creating a scenario in which the search area is a public space. The starting number of crows and their location in each subsequent iteration are defined as follows for the purpose of initializing the optimization problem:

15 $$\chi^{ci,It} = \left[ {\chi_{1}^{ci,It} ,\chi_{2}^{ci,It} ,\chi_{3}^{ci,It} ,............\chi_{md}^{ci,It} } \right]$$

The variables of the of the considered DS operational planning problem are represented in Eq. (15) in which $ci$ and $md$ represent respectively, the crow number and the maximal dimension. The crow is saved at its best current position at a time, after exploiting or exploring a number of places and return into the scanning of areas for more food sources to supplement their existing food supply spots which were their previously best position. To enhance the search and improve the exploitation and exploration process the CSO was modified to achieve better optimization outcomes. The modification includes two parameters namely the potential of knowing (PK) and flight duration (LF). The modified crow search optimization (MCSO) starts with a random solutions group and searches memory to find the best solution in the available space, then produces a new scenario making use of PK. Two scenarios of the CSO are developed for the considered distribution system. In scenario one, a particular crow number $(f)$ is not aware that they are being pursued by the crow number $(e)$ and this result in crow $(e)$ attaining an area where the crow food source is stashed. During this scenario, the new crow number $(e)$ position can be represented by Eq. (16) as follows:

16 $$\chi^{f,It + 1} = \chi^{f,It} + Rand_{0}^{1} \times LF^{f,It} \times (M^{e,It} - \chi^{f,It} )$$

where, $It$ is the crow population iteration, $Rand_{0}^{1}$ is the random number generated between 0 and 1, $LF^{f,It}$ is the flight duration of the crow population iteration and $M^{e,It}$ is the best position of the crow population at a given iteration.

In step 2, the crow number $(f)$ senses that the crow number $(e)$ is approaching near them. In reaction, crow number $(f)$ employs cunning techniques to keep crow number $(e)$ from snatching their food by pretending and just patrolling around in the search area. The two scenarios are represented in Eq. (17) as follows:

17 $$\chi^{f,It + 1} = \left\{ {\begin{array}{*{20}l} {\chi^{f,It} + Rand_{0}^{1} \times LF^{f,It} \times \left( {M^{e,It} - \chi^{f,It} } \right)} \hfill & {if} \hfill & {Rand \ge PK^{f,It} } \hfill \\ {RP} \hfill & {} \hfill & {Otherwise} \hfill \\ \end{array} } \right.$$

where, $RP$ is the random position generated for the crow.

Additionally, better performance of the CSO can be achieved through the application of the inertia weight reduction on the conventional CSO. This method contributes to the enhancement of the conventional CSO in both local and global exploration. The inertia weight $\zeta$ undergoes non-linear variations from a maximum value $\zeta_{mx}$ to minimum value $\zeta_{mx}$ as shown in Eq. (18). A greater value of $\zeta$ is acquired for the global optimal search capability of the crow population, while a smaller value of $\zeta$ is produced for the local optimal search capability.

18 $$\zeta = \zeta_{mn} + \left( {0.5 + 0.5 \times \cos \left( {\frac{\pi \times It}{{It_{mx} }}} \right)} \right)^{\sigma } \times \left( {\zeta_{mx} - \zeta_{mn} } \right)$$

where, $It_{mx}$ is the maximum number iteration and $\sigma$ is a constant.

The comprehensive MCSO including the inertia weight is expressed by Eq. (19) as follow:

19 $$\chi^{f,It + 1} = \left\{ \begin{gathered} \zeta \times \chi^{f,It} + Rand_{0}^{1} \times LF^{f,It} \times \left( {M^{e,It} - \chi^{f,It} } \right)\,\,\,\,\,\,\,\,\,\,\,\,\,\,\,\,\,\,if\,\,Rand \ge PK^{f,It} \hfill \\ RP\,\,\,\,\,\,\,\,\,\,\,\,\,\,\,\,\,\,\,\,\,\,\,\,\,\,\,\,\,\,\,\,\,\,\,\,\,\,\,\,\,\,\,\,\,\,\,\,\,\,\,\,\,\,\,\,\,\,\,\,\,\,\,\,\,\,\,\,\,\,\,\,\,\,\,\,\,\,\,\,\,\,\,\,\,\,Otherwise \hfill \\ \end{gathered} \right.$$

**Fig. 1:**
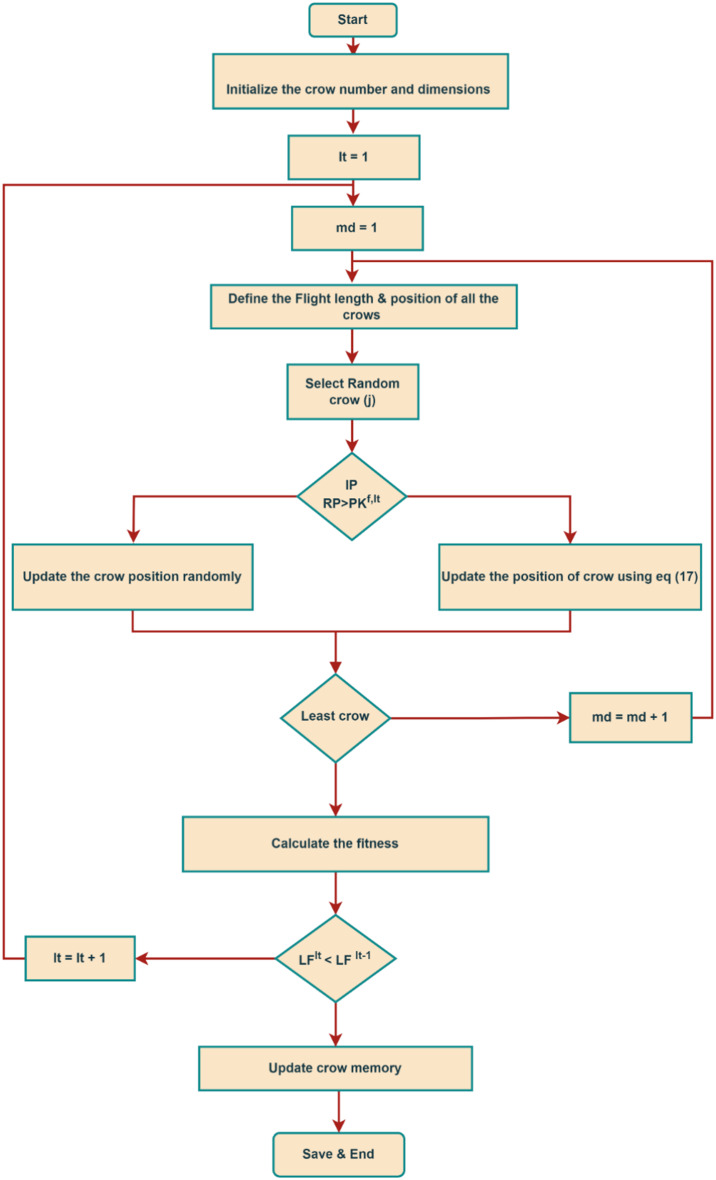
Operation flow chart of the considered multi-objective optimization problem.

**Fig. 2:**
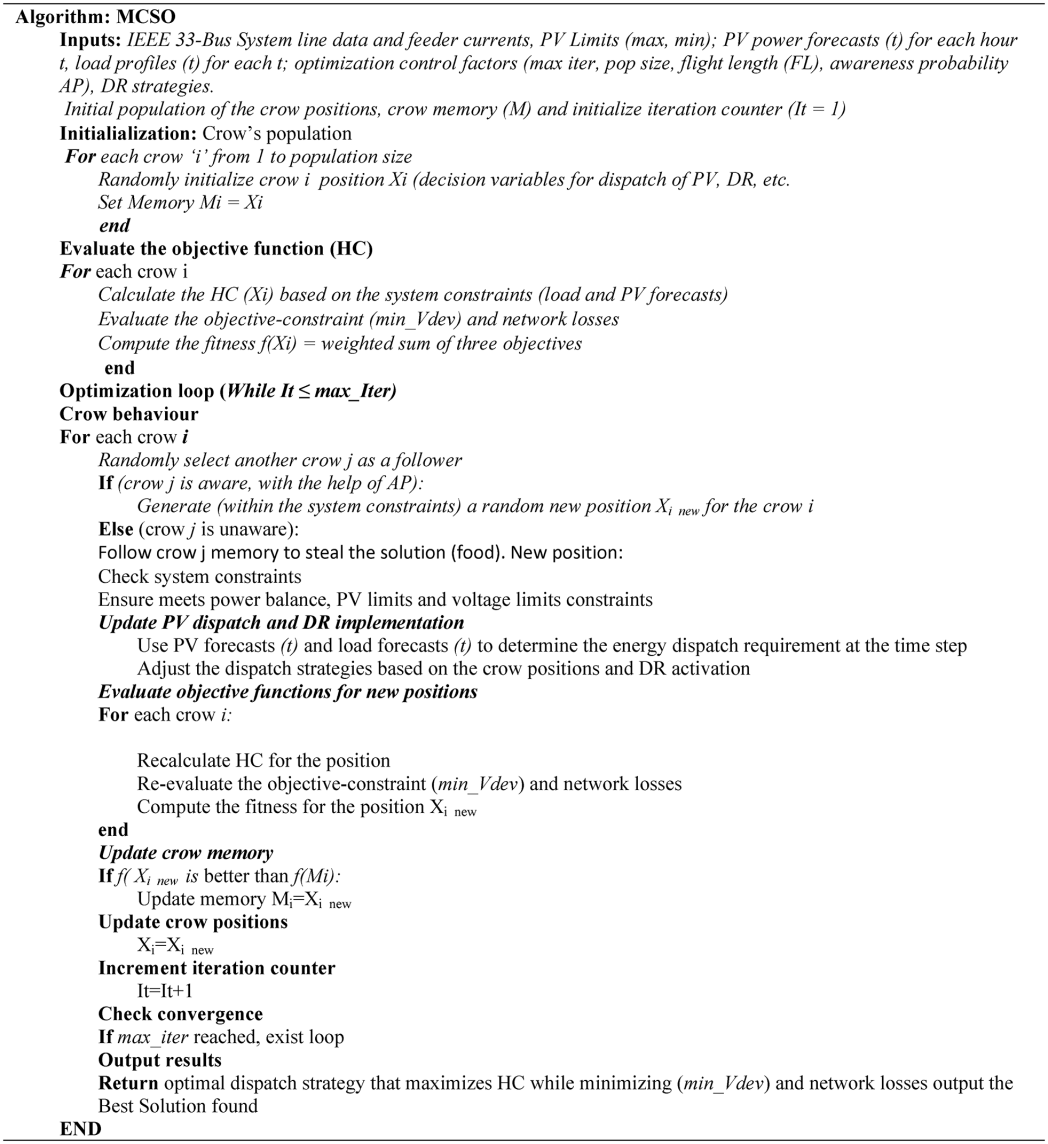
Pseudocode for the MCSO.

To develop the multi-objective operational planning problem with MCSO, Figs. 1 and 2 represents the flow chart and the pseudocode as follows:

- Assign the input data. These data consist of distribution system line data, PV units upper and lower limits, hourly PV power forecasts, hourly load profiles, and optimization algorithm control factors.
- Initialize each individual and decision variable in accordance with Eq. (15) as previously mentioned.
- The forecasted solar PV system profiles are assigned to the particular location of the DS to determine the best possible operating schedule for the resources under consideration.
- The optimization variables comprise of the capacity of the photovoltaic system and the regulation of the variable load in response to uncertain real-time pricing.
- Equations (5) to (14) illustrate how to plan the variable load and solar systems as efficiently as possible while keeping the network nodal voltage stable using the data assigned to the distribution system.
- The node voltage and power loss of the distribution system is determined by using the Newton–Raphson load flow method.
- In order to provide a best set of solutions, carry out the two scenarios of the crow search optimization algorithm as previously stated in Eq. (17).
- Determine the framed multi objective of distribution system and execute the selection process for the best fitness and population for upcoming generation.
- Save the best population and fitness of the considered multi-objective operational planning problem of distribution system.

## Validation of MCSO

**Fig. 3:**
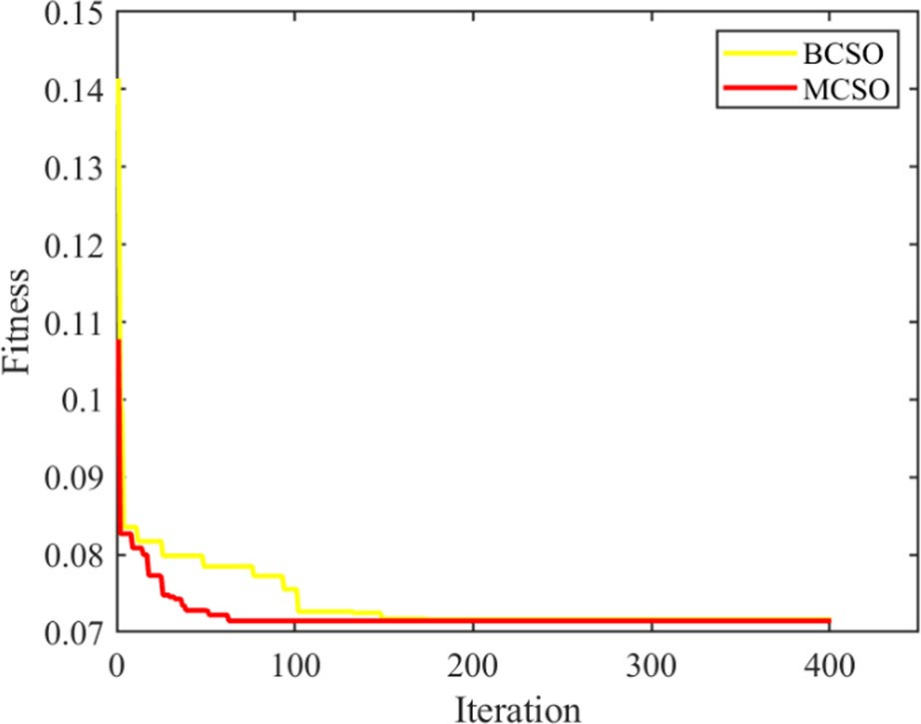
Convergence Plot of MCSO and BCSO.

The proposed MCSO model is validated before being used to the developed operational planning model. This validation uses a single objective function to minimize network power losses and simulates optimal deployment of DGs in a 33-bus network. The mathematical nature for both the optimization problem are same and is mixed integer nonlinear and non-convex. Table 1 shows that the best fitness for MCSO is better and comparable than the basic crow search optimization (BCSO) and other established OA. To account for the randomness of these meta-heuristic procedures, 50 independent trials were conducted. Table 2 compares a few parameters of described approaches throughout 400 iterations and 50 population sizes. This table includes characteristics such as worst, mean, and highest fitness, as well as the standard deviation of fitness obtained after 50 runs and the time required to execute the program in a single run. Table 2 shows that the MCSO outperforms BCSO. Figure 3 shows the convergence characteristics of basic crow search optimization and MCSO for the value of best fitness in a single run. The computation time for MCSOA is less than the basic and standard deviation is also improved to 0.00210.

**Table 1:** Comparison of MCSO with some existing optimizations.

Optimization Algorithm (OA) methods	Locations {L1,L2, L3}, Size {S1,S2,S3} (MW) [L1,L2,L3; S1,S2,S3]	Power loss (MW)
Base case	DG not installed	0.2027
GA ^39^	[11,29,30;1.500,0.423,1.071]	0.1063
PSO ^39^	[08,13,32;1.177,0.982,0.830]	0.1053
Teaching Learning Based OA (TLBO) ^40^	[12,28,30;1.183,1.191,1.186]	0.1246
Improved analytical OA (IA) ^41^	[06,12,31;0.900,0.900,0.720]	0.0811
Improved Water evaporation method (IWEO) ^42^	[14,24,30;0.757,1.096,1.080]	0.0714
BCSO	[13,24,30;0.802,1.090,1.054]	0.0721
MCSO	[15,24,30;0.754,1.099,1.071]	0.0714

**Table 2:** Comparison of MCSO with BCSO.

OA Method	Worst fitness (MW)	Best fitness (MW)	Mean fitness (MW)	CPU time (Sec.)	Standard deviation
BCSO	0.0793	0.0721	0.0751	70.16	0.00234
MCSO	0.0786	0.0714	0.0748	69.27	0.00210

## Results discussion

Seven distinct case studies are used to assess the effectiveness of the standard 33-bus distribution system planning and operation optimization model, and each case study is examined both with and without demand response. Investigating the effects of demand response on the hosting capacity of photovoltaic on the distribution system planning and operation problem involves taking into consideration different involvement levels of the consumers. The PV generation model used in the case studies has a rated illumination intensity of 1000 Wm^–2^, with corresponding form and scale indices of 1.8 and 5.5 respectively. A single-objective optimization model with maximizing solar PV HC, minimizing network losses and minimize node voltage deviation for DS is represented by case 1, case 2, and case 3 respectively. Case 4 represents the multi-objective optimization model that optimize the HC and network losses simultaneously, while case 5 considers HC optimization nodal voltage deviation. Furthermore, case 6 represents the multi-objective model that optimize network losses and node voltage deviation. The case 7 is developed to optimize all three conflicting objectives of the DS namely: maximize of HC of photovoltaic, minimize node voltage deviation and, minimize network losses simultaneously. In all the developed case studies, the suggested optimization is expressed as a mixed integer non-linear programming model.

Case 1: Single objective: maximize HC of PV for the distribution system.

Case 2: Single objective: minimize network losses.

Case 3: Single objective: minimize V_Dev_.

Case 4: Multi-objective considering maximize HC and minimize network losses.

Case 5: Multi-objective considering maximize HC and minimize V_Dev_.

Case 6: Multi-objective considering minimize network losses and minimize V_Dev_.

Case 7: Multi-objective considering maximize HC of PV, minimize network losses and minimize V_Dev_.

Table 3 presents the summary of consolidated results focusing on solar photovoltaic capacity (CapPPV1 & CapPPV2), on solar photovoltaic hosting capacity (HC), the amount of network losses (loss) and voltage deviation (V_Dev_), particularly for cases 1, 2 and 3. Table 4 on the other hand presents similarly the consolidated results for analysis of cases 4,5,6 and 7 under the same consideration.

**Table 3:** Summary of cases 1, 2 and 3 single-objective optimization considering HC, network losses and V_Dev_ without and with DR consideration.

Cases	C-1		C-2		C-3	
	Objective—maximize HC		Objective—minimize losses		Objective—minimize Voltage deviation	
	Without DR	With DR	Without DR	With DR	Without DR	With DR
CapPPV1 [kW]	348.49	2771.48	804.68	934.67	1244.89	1471.44
CapPPV2 [kW]	2448.57	630.94	1038.43	1200.01	1510.00	1851.27
HC [kW]	2797.07	3402.43	1843.11	2134.69	2754.90	3322.71
Loss [kWh]	1653.19	1913.215	1418.50	1197.10	1490.02	1316.40
V_Dev_ [p.u.]	0.705	0.705	0.719	0.558	0.676219882	0.498108

**Table 4:** Summary of cases 4, 5, 6 and 7 for multi-objective optimization considering combination of HC, network losses and V_Dev_ without and with DR consideration.

Cases	C-4		C-5		C-6		C-7	
	Without DR	With DR	Without DR	With DR	Without DR	With DR	Without DR	With DR
CapPPV1 [kW]	1117.50	1347.22	1239.67	1433.31	1226.11	1388.99	1254.19	1490.24
CapPPV2 [kW]	1636.96	1974.5	1515.28	1890.01	1400.19	1516.05	1500.63	1832.06
HC [kW]	2754.47	3321.72	2754.95	3323.32	2626.30	2905.04	2754.83	3322.31
Loss [kWh]	1487.71	1311.43	1490.08	1317.76	1471.68	1248.63	1489.94	1314.86
V_Dev_ [p.u.]	0.6782914	0.50136	0.6762209	0.49816403	0.678743042	0.50848	0.6762233	0.49827

### Case 1: Single objective: maximize HC of PV for the distribution system

The suggested model is examined in this case study with the objective of optimizing the hosting capacity of photovoltaic for distribution system. Table 3 presents the case study simulation results. With no DR strategy implemented, the optimal size of the photovoltaic is 2797.07 kW and it can be observed that the size increases by 21.64% to 3321.72, when successfully applying DR. This can be justified by the fact that power utilities can smooth demand peaks and valleys by providing incentives to customers to modify their electricity usage and shift the consumption toward the period of lower demands. The variable solar generation can be more effectively integrated into the grid due to this load control flexibility. When solar generation is high, utilities can indeed incentivize customers to utilize more electricity or store more energy resulting in the rise of the solar array effective hosting capacity. Case 1, alongside, reveals that the network losses rise from 1653.19 to 1913.215 kW representing a 15.73% rise when demand response is incorporated with the aim of expanding photovoltaic hosting capacity clearly showing that the earlier objective and the objective of minimizing network energy losses of a distribution system are indeed two conflicting objectives in nature. The deviation of the node voltage for this case remained however unchanged at 0.705 p.u. for the change that was observed in the HC.

Moreover, from the observation of the results displayed in Table 5, it can be confirmed that when DR is implemented, photovoltaic system 1 (PPV1) supplies more power to the distribution system between 11:00 and 14:00 as compared to the scenario without demand response. This shows that with demand response, photovoltaic resource is optimally utilized. It is important to note that at this period of time, the burden of responding to load demand lies on the photovoltaic capacity as the grid demand appears to be zero both without or with demand response. It shall also be noted that with the demand response implementation, the node voltage deviation had improved. This can be seen through the improvement from 0.9137 to 0.9248 p.u. of the minimum voltage at node 18 which is the weakest node of the scenario as per Table 6.

**Table 5:** PV capacity and grid demand for single-objective considering HC maximization without and with DR.

Without DR				With DR		
Time [h]	PPV1 [MW]	PPV2 [MW]	Grid demand [MWh]	PPVDR1 [MW]	PPVDR2 [MW]	Grid demand DR [MWh]
1	0	0	1.045098	0	0	0.833778556
2	0	0	1.010469	0	0	0.806230161
3	0	0	1.158346	0	0	0.923830216
4	0	0	1.02386	0	0	0.816883917
5	0	0	1.22312	0	0	0.975308852
6	0.014183	0.099652	1.565887	0.112794	0.025678	1.201586281
7	0.016972	0.119245	1.655347	0.13497	0.030727	1.264427949
8	0.082165	0.577301	1.810695	0.653434	0.148758	1.817172887
9	0.136514	0.959159	1.549294	1.085651	0.247154	1.646821274
10	0.256345	1.8011	0.384946	2.038625	0.464104	0.10850736
11	0	1.915588	0	2.01271	0.327237	0
12	0	2.218389	0	2.081757	0.607261	0
13	0	2.153378	0	2.066975	0.547153	0
14	0	1.991435	0	2.030052	0.397392	0
15	0.210684	1.480282	0.636885	1.675499	0.381436	0.227516641
16	0.094695	0.665339	1.633283	0.753082	0.171443	1.61144457
17	0.014133	0.099302	3.300653	0.112397	0.025588	3.757037109
18	0.006455	0.045351	3.305034	0.051332	0.011686	3.147094148
19	0.00372	0.026138	3.882337	0.029585	0.006735	3.379262963
20	0	0	2.98136	0	0	3.03686378
21	0	0	3.100546	0	0	3.011912637
22	0	0	2.763749	0	0	3.201652776
23	0	0	2.335998	0	0	1.95517736
24	0	0	1.68231	0	0	1.343030844

**Table 6:** Node voltage profile for single-objective considering maximizing HC with and without DR.

Without DR			With DR	
Nodes	Mean voltage [p.u]	Minimum voltage [p.u]	Mean voltage DR [p.u]	Minimum voltage DR [p.u]
1	1	1	1	1
2	0.998685558	0.997053632	0.998754554	0.997157398
3	0.992678684	0.983071446	0.993118081	0.983691363
4	0.989919049	0.975670902	0.990634509	0.976829112
5	0.987271904	0.968359441	0.988275831	0.970067643
6	0.980200103	0.950147314	0.981816317	0.953287655
7	0.97831083	0.946670634	0.980532233	0.950112828
8	0.975783367	0.941849294	0.980500611	0.946086701
9	0.972547044	0.935614093	0.980851031	0.941189179
10	0.969566296	0.929832384	0.981523871	0.936774371
11	0.969134602	0.928977795	0.981785076	0.936160447
12	0.968384324	0.9274796	0.982353116	0.93509839
13	0.965338753	0.921411096	0.984470552	0.930910897
14	0.964209313	0.919160745	0.985256825	0.929430024
15	0.963430744	0.917750303	0.984493102	0.928136184
16	0.962676784	0.916384215	0.98375361	0.926922583
17	0.961559725	0.91435953	0.982658865	0.925249346
18	0.961225184	0.913753217	0.982331575	0.924827194
19	0.998381954	0.996525529	0.99845086	0.996599088
20	0.996326868	0.99294963	0.996395038	0.992522721
21	0.995922217	0.992245475	0.995990236	0.991677168
22	0.995556132	0.991608372	0.995624008	0.990912092
23	0.990638602	0.979487621	0.99107851	0.979922063
24	0.986844504	0.972820537	0.987285539	0.972911973
25	0.984950299	0.969490114	0.985391822	0.969409929
26	0.979867843	0.948255867	0.980932159	0.9514794
27	0.979480467	0.945745816	0.979771496	0.949088492
28	0.977003185	0.934510153	0.97441082	0.938545995
29	0.97540764	0.926446213	0.970602468	0.931010869
30	0.975305225	0.922984472	0.969098341	0.927796653
31	0.973028613	0.918829845	0.966809084	0.923581094
32	0.972527823	0.917915895	0.966305493	0.922672373
33	0.972372642	0.917632666	0.966149224	0.92244048

### Case 2: Single-objective minimize network energy losses

The developed optimization model for this case is framed to minimize the network losses with and without the impact of demand response. From Table 3 consolidated results, the network energy losses are reduced by 15.58% with the incorporation of demand response. Moreover, the losses for this case are reduced to 37.42% as compared to case 1. It is also confirmed from this investigation results that when the minimization of network losses is considered as the main objective function, the hosting capacity of photovoltaic is decreased by 37.27% as compared to case 1. This establishes once more the conflicting nature of the hosting capacity and the network losses objective functions. From Table 7 results, it is evident that when the focus is the minimization of network losses for the distribution system, the demand taken from the grid is no longer zero as in case1 at the considered period meaning from 11:00 to 14:00 h. This is consequent to the reduction of the hosting capacity of photovoltaic compare to case 1 as stated earlier. From Table 3, it can be recorded that the voltage deviation in this case is reduced to 0.558 p.u. from 0. 719 p.u. implying that reduced losses result in a more consistent voltage profile throughout the network, with less variance across the nodes. Indeed, the nodes are less likely to encounter notable voltage dips or spikes as a result of local losses, which assist in lowering voltage deviations. Moreover, the analysis of hosting capacity is intended to avert situations in which an overabundance of solar integration could result in voltage violations or instability.

**Table 7:** PV capacity and grid demand for single-objective of minimizing network losses without and with DR.

Without DR				With DR		
Time [h]	PPV1 [MW]	PPV2 [MW]	Grid demand [MWh]	PPVDR1 [MW]	PPDR2 [MW]	Grid demand DR [MWh]
1	0	0	1.045098	0	0	1.257644
2	0	0	1.010469	0	0	1.215854
3	0	0	1.158346	0	0	1.394373
4	0	0	1.02386	0	0	1.232013
5	0	0	1.22312	0	0	1.472619
6	0.032749	0.042262	1.606091	0.038039	0.048838	1.829434
7	0.039188	0.050571	1.703536	0.045519	0.05844	1.871411
8	0.18972	0.244832	2.04165	0.22037	0.282929	1.874369
9	0.315212	0.406777	1.926134	0.366134	0.470073	1.815765
10	0.591902	0.763842	1.056898	0.687523	0.882699	1.296784
11	0.505185	0.782951	0.582487	0.586589	0.901734	0.761112
12	0.565816	0.870917	0.720675	0.659598	1.007903	0.927825
13	0.552845	0.852086	0.691097	0.643975	0.985167	0.892131
14	0.520423	0.805046	0.617202	0.604935	0.928392	0.802981
15	0.48647	0.627784	1.197447	0.565059	0.725469	1.454461
16	0.218653	0.282168	1.897573	0.253976	0.326075	1.865766
17	0.032634	0.042114	3.342618	0.037906	0.048667	2.619492
18	0.014904	0.019233	3.324235	0.017312	0.022226	2.6207
19	0.00859	0.011085	3.893597	0.009977	0.01281	3.071341
20	0	0	2.98136	0	0	2.364814
21	0	0	3.100546	0	0	2.458407
22	0	0	2.763749	0	0	2.197
23	0	0	2.335998	0	0	2.001465
24	0	0	1.68231	0	0	1.844553

It is appropriate to specify that unlike for the objective of loss reduction, hosting capacity does not automatically optimize or minimize voltage deviations. If solar energy is not properly controlled or if there are local restrictions affecting voltage levels, voltage variations may still happen. Table 8 displays the profile of the node voltage deviation under the objective of network losses minimization both with and without demand response implementation.

**Table 8:** Node voltage profile for single-objective of minimizing network losses without and with DR.

Without DR			With DR	
Nodes	Mean voltage [p.u]	Minimum voltage [p.u]	Mean voltage DR [p.u]	Minimum voltage DR [p.u]
1	1	1	1	1
2	0.99857085	0.997046951	0.998615407	0.997669748
3	0.991949481	0.983029027	0.99223235	0.98662557
4	0.988733414	0.975602046	0.989190902	0.980796221
5	0.985610159	0.968263063	0.986249407	0.975039976
6	0.977520446	0.949990815	0.978561506	0.960707912
7	0.975788319	0.946520544	0.976874695	0.957982826
8	0.973864604	0.941722554	0.975093846	0.954223693
9	0.971504108	0.935521578	0.972939487	0.949374486
10	0.969412385	0.929774554	0.971055306	0.944883349
11	0.969148355	0.928926479	0.970829534	0.944221522
12	0.968717266	0.927440691	0.970470842	0.943062913
13	0.966924231	0.921421022	0.968963323	0.938370381
14	0.966257583	0.919188673	0.968402357	0.936630541
15	0.965480527	0.917778274	0.96763145	0.935524465
16	0.964728032	0.916412229	0.966884923	0.934453276
17	0.963613145	0.914387606	0.96577893	0.932865984
18	0.963279254	0.913781311	0.965447705	0.932390632
19	0.99826721	0.996518845	0.998311879	0.997247884
20	0.996211883	0.992942921	0.996257381	0.994391886
21	0.995807184	0.992238762	0.99585285	0.993829515
22	0.995441055	0.991601655	0.995486876	0.993320722
23	0.989907858	0.979445043	0.99019417	0.983775783
24	0.986110893	0.972777665	0.986403736	0.978475144
25	0.984215254	0.969447094	0.984511483	0.97582816
26	0.976760382	0.948078047	0.977856714	0.959210296
27	0.975773744	0.945538165	0.976947173	0.957222603
28	0.971057201	0.934189888	0.972526007	0.948336407
29	0.967741757	0.926040369	0.969435135	0.941956964
30	0.966553154	0.922525287	0.968382845	0.939210125
31	0.96425668	0.918368569	0.966102979	0.935947975
32	0.963751521	0.917454158	0.96560148	0.935230379
33	0.963594986	0.917170787	0.965446082	0.935008008

The results tabulated above show that minimum node voltage at node 18 improved from 0.9137 to 0.9323 p.u. due to the impact of demand response.

### Case 3: Single objective minimize the V_Dev_

Case 3 is developed with the goal of minimizing the node voltage deviation as the objective function. The result outcomes for this case are shown in Tables 3, 9 and 10. From Table 3, it shall be observed that without DR implementation, the node voltage deviation had decreased from 0.70553 p.u. in case 1 to 0.676222 p.u. in this case 3 since the model was developed to minimize the node voltage deviation. Focusing at case 3, the implementation of demand response leads to a further decrease from 0.6762 to 0.4981 p.u. Although the network losses in this case 3 were not targeted, it can be established that the losses have decreased from 1490.40 kWh without demand response to 1316.40 kWh when demand response is applied. Furthermore, the hosting capacity of photovoltaic has decreased in both scenarios with or without demand response when case 3 is compared to case 1.

**Table 9:** Capacity of PV and grid demand for single-objective of minimizing V_Dev_ without and with DR.

Without DR				With DR		
Time [h]	PPV1 [MW]	PPV2 [MW]	Grid demand [MWh]	PPVDR1 [MW]	PPVDR2 [MW]	Grid demand DR [MWh]
1	0	0	1.045098	0	0	1.257644
2	0	0	1.010469	0	0	1.215854
3	0	0	1.158346	0	0	1.394373
4	0	0	1.02386	0	0	1.232013
5	0	0	1.22312	0	0	1.472619
6	0.050665	0.061454	1.567384	0.059884	0.075343	1.685119
7	0.060626	0.073537	1.657113	0.071659	0.090157	1.71372
8	0.29351	0.356015	1.817844	0.346922	0.436476	1.613158
9	0.487654	0.591503	1.55984	0.576395	0.725185	1.655339
10	0.915711	1.110719	0.401502	1.082348	1.361746	0.481838
11	0.627832	1.261165	0	0.7313	1.546194	0
12	0.729411	1.453329	0	0.851825	1.781788	0
13	0.694166	1.425751	0	0.809506	1.747977	0
14	0.734241	1.228096	0	0.860358	1.505651	0
15	0.752601	0.912874	0.65185	0.889557	1.119187	0.781377
16	0.33827	0.410307	1.641417	0.399827	0.503038	1.591893
17	0.050487	0.061238	3.301901	0.059674	0.075078	2.567711
18	0.023057	0.027968	3.305617	0.027253	0.034288	2.597001
19	0.013289	0.016119	3.882651	0.015707	0.019762	3.057474
20	0	0	2.98136	0	0	2.364814
21	0	0	3.100546	0	0	2.458407
22	0	0	2.763749	0	0	2.193724
23	0	0	2.335998	0	0	1.856657
24	0	0	1.68231	0	0	1.70513

**Table 10:** Node voltage profile for single-objective of minimizing V_Dev_ without and with DR.

Without DR			With DR	
Nodes	Mean voltage [p.u]	Minimum voltage [p.u]	Mean voltage DR [p.u]	Minimum voltage DR [p.u]
1	1	1	1	1
2	0.998685575	0.997053491	0.998764031	0.997677962
3	0.992678701	0.983070549	0.99317734	0.986677719
4	0.989918969	0.975669445	0.990727456	0.980880898
5	0.98727164	0.968357399	0.988403181	0.975158515
6	0.980208425	0.950144274	0.982047002	0.960900416
7	0.978559752	0.946681352	0.980461054	0.958183761
8	0.976949473	0.9419075	0.979057708	0.954453132
9	0.975040382	0.935741648	0.977446562	0.949645343
10	0.973406447	0.930030043	0.976113285	0.945196017
11	0.9732285	0.929188541	0.97599106	0.944541973
12	0.972960995	0.927715231	0.975829082	0.943398149
13	0.971809625	0.921744737	0.975093266	0.938763815
14	0.971380503	0.919530524	0.974818112	0.93704544
15	0.970607495	0.918120654	0.974052908	0.935939858
16	0.969858921	0.916755122	0.973311904	0.934869146
17	0.96874985	0.914731261	0.972214092	0.933282564
18	0.968417701	0.914125195	0.971885313	0.932807424
19	0.998381971	0.996525388	0.99846055	0.997256102
20	0.996326885	0.992949488	0.99640637	0.994400127
21	0.995922234	0.992245334	0.996001901	0.993837761
22	0.995556148	0.99160823	0.995635983	0.993328972
23	0.990638616	0.97948672	0.99114135	0.983828085
24	0.986844512	0.97281963	0.987354993	0.978527733
25	0.984950305	0.969489204	0.985464793	0.975880892
26	0.979614129	0.948238855	0.981565916	0.9594126
27	0.978859501	0.945709239	0.980969529	0.957438605
28	0.975012261	0.934399921	0.977722428	0.948604154
29	0.972362254	0.926279953	0.975531737	0.942263983
30	0.97159171	0.922783113	0.975045019	0.939541525
31	0.969306752	0.918627569	0.972782347	0.936280536
32	0.968804127	0.917713417	0.972284627	0.935563195
33	0.968648376	0.917430126	0.972130398	0.935340903

Table 9 results show the optimal utilisation of photovoltaic capacity with the minimization of node voltage deviation as the objective function. Both with or without demand response implementation, the solar PV capacity had increased to cater exclusively for the load demand between 11:00 and 14:00 h, period during which grid demand is null.

Finally from Table 10 displayed results, it is argued that the optimization framework under this case has achieved the targeted aim since the voltage at the weakest node, is improved from 0.9141 p.u to 0.9328 p.u when successfully implementing demand response.

### Case 4: Multi-objective considering HC of PV and network losses

This case study aims to simultaneously optimize hosting capacity of photovoltaic and network losses in a multi-objective developed framework and results from Tables 3, 4, 11 and 12 are discussed. With reference to this case, the results from Table 4 show that although the hosting capacity had improved to 3321.72 kW after DR implementation from 2754.47 kW, the hosting capacity has slightly reduced globally in case 4 compared to case 1 by respectively 1.52% before DR and by 2.37% after DR implementation. However, this reduction did not affect load shifting as from Table 11 results the grid demand is zero between 11:00 and 14:00 h.

**Table 11:** PV capacity and grid demand for multi-objective optimization considering HC and network losses with and without DR.

Without DR				With DR		
Time [h]	PPV1 [MW]	PPV2 [MW]	Grid demand [MWh]	PPVDR1 [MW]	PPVDR2 [MW]	Grid demand DR [MWh]
1	0	0	1.045098	0	0	1.257644
2	0	0	1.010469	0	0	1.215854
3	0	0	1.158346	0	0	1.394373
4	0	0	1.02386	0	0	1.232013
5	0	0	1.22312	0	0	1.46445
6	0.04548	0.066621	1.567433	0.054829	0.080358	1.687245
7	0.054422	0.079719	1.657174	0.065609	0.096157	1.724907
8	0.263475	0.385947	1.818055	0.317636	0.465528	1.644425
9	0.437752	0.641233	1.56	0.527737	0.773454	1.517114
10	0.822007	1.204101	0.400945	0.99098	1.452385	0.474393
11	0.709775	1.178879	0	0.855324	1.421386	0
12	0.819931	1.362391	0	0.988743	1.643917	0
13	0.796327	1.323056	0	0.960139	1.596189	0
14	0.737419	1.224918	0	0.888788	1.47718	0
15	0.675588	0.989622	0.651594	0.814463	1.193681	0.774464
16	0.303655	0.444803	1.641611	0.366075	0.536521	1.613595
17	0.04532	0.066387	3.301997	0.054637	0.080076	2.567808
18	0.020698	0.030319	3.305662	0.024953	0.036571	2.597047
19	0.011929	0.017474	3.882682	0.014381	0.021077	3.057504
20	0	0	2.98136	0	0	2.364814
21	0	0	3.100546	0	0	2.458407
22	0	0	2.763749	0	0	2.193724
23	0	0	2.335998	0	0	1.937452
24	0	0	1.68231	0	0	1.718971

**Table 12:** Node voltage profile with multi-objective considering HC and network losses without and with DR.

Without DR			With DR	
Nodes	Mean voltage [p.u]	Minimum voltage [p.u]	Mean voltage DR [p.u]	Minimum voltage DR [p.u]
1	1	1	1	1
2	0.998685592	0.997053466	0.998764062	0.99767794
3	0.992678814	0.983070391	0.993177507	0.986677576
4	0.98991915	0.97566919	0.990727664	0.980880666
5	0.987271891	0.968357042	0.9884034	0.975158191
6	0.980208941	0.950143655	0.982047375	0.960899861
7	0.978558131	0.946678776	0.980466661	0.958181386
8	0.976941128	0.941898172	0.979086673	0.954444341
9	0.975022131	0.935722463	0.977508849	0.949627203
10	0.973378227	0.930000893	0.976209605	0.94516842
11	0.973198436	0.929157531	0.976093871	0.944512607
12	0.972927425	0.927680684	0.97594424	0.943365418
13	0.971762201	0.921696261	0.97525665	0.938717846
14	0.971327988	0.919476911	0.974999429	0.936994589
15	0.970554869	0.918066958	0.974234337	0.935888946
16	0.969806188	0.916701346	0.973493445	0.934818177
17	0.968696957	0.914677365	0.972395798	0.933231508
18	0.96836476	0.914071263	0.972067068	0.932756341
19	0.998381989	0.996525363	0.998460585	0.997256079
20	0.996326903	0.992949463	0.996406426	0.994400104
21	0.995922252	0.992245309	0.996001961	0.993837739
22	0.995556166	0.991608206	0.995636047	0.993328949
23	0.990638728	0.979486562	0.991141549	0.983827942
24	0.986844625	0.972819471	0.987355251	0.978527589
25	0.984950418	0.969489045	0.985465063	0.975880747
26	0.979616629	0.948240124	0.981559649	0.959413838
27	0.978864774	0.945713153	0.98095393	0.957442354
28	0.975028104	0.934413786	0.977672153	0.948617333
29	0.972385917	0.926301389	0.975454216	0.942284332
30	0.971620172	0.922809294	0.974949976	0.939566375
31	0.969335391	0.918653869	0.972687396	0.936305474
32	0.968832805	0.917739743	0.972189697	0.935588152
33	0.968677067	0.917456461	0.972035477	0.935365866

This shows that the solar PV increased capacity is fully catering for the load demand with the help of DR implementation under the considered objectives. Furthermore, Table 4 network losses results confirm that in case 4, losses reduced from 1487.71 kWh without DR down to 1311.43 kWh with DR implementation, situation quasi opposite to case 1 in which losses increased from no-DR to DR implementation.

Additionally, a staggering 31.45% loss reduction is observed from case 4 as compared to case 1 when both cases are implementing DR under their specific optimization frames. This shows the effectiveness of the developed multi-objective approach compared to a single objective approach. As per Table 4, and 12 results respectively, it is noted that the with the implementation of DR node voltage deviation is improved to 0.50136 p.u. in case 4 from 0.701 p.u. and the minimum voltage at node 18 has improved from 0.9248 p.u. in case 1 to 0.9327 p.u in case 4.

### Case 5: Multi-objective considering simultaneously HC of PV and V_Dev_

This case is a multi-objective formulation model developed with consideration of solar PV HC and node voltage deviation simultaneously. Results from Tables 3, 4, 13 and 14 are concerned with this case. It is seen from Table 4 results that the voltage deviation for this case is 0.6762 p.u. and 0.4982 p.u. without and with DR implementation.

**Table 13:** PV Capacity and grid demand for the multi-objective optimization model considering HC and V_Dev_.

Without DR				With DR		
Time [h]	PPV1 [MW]	PPV2 [MW]	Grid demand [MWh]	PPVDR1 [MW]	PPVDR2 [MW]	Grid demand DR [MWh]
1	0	0	1.045098	0	0	1.257644
2	0	0	1.010469	0	0	1.215854
3	0	0	1.158346	0	0	1.394373
4	0	0	1.02386	0	0	1.232013
5	0	0	1.22312	0	0	1.472619
6	0.050452	0.061669	1.567383	0.058333	0.07692	1.68479
7	0.060372	0.073794	1.657112	0.069802	0.092043	1.713818
8	0.292278	0.357259	1.817835	0.337932	0.445609	1.616874
9	0.485606	0.593569	1.559815	0.561459	0.74036	1.648901
10	0.911867	1.114598	0.401416	1.054302	1.39024	0.481041
11	0.623465	1.26557	0	0.699405	1.578548	0
12	0.724382	1.458405	0	0.815124	1.819071	0
13	0.689239	1.43073	0	0.773552	1.784553	0
14	0.729954	1.232385	0	0.828993	1.537156	0
15	0.749442	0.916062	0.651789	0.866506	1.142606	0.780791
16	0.33685	0.41174	1.641404	0.389467	0.513564	1.590715
17	0.050275	0.061452	3.301902	0.058128	0.076649	2.567701
18	0.022961	0.028065	3.305617	0.026547	0.035006	2.596997
19	0.013233	0.016175	3.882651	0.0153	0.020175	3.057473
20	0	0	2.98136	0	0	2.364814
21	0	0	3.100546	0	0	2.458407
22	0	0	2.763749	0	0	2.193724
23	0	0	2.335998	0	0	1.856657
24	0	0	1.68231	0	0	1.708749

**Table 14:** Node voltage profile in multi-objective framework considering HC and V_Dev_.

Without DR			With DR	
Nodes	Mean voltage [p.u]	Minimum Voltage [p.u]	Mean Voltage DR [p.u]	Minimum Voltage DR [p.u]
1	1	1	1	1
2	0.998685579	0.99705349	0.998764065	0.997677962
3	0.992678729	0.983070545	0.993177561	0.986677715
4	0.989919013	0.975669439	0.990727815	0.980880892
5	0.987271701	0.968357391	0.988403686	0.975158506
6	0.980208523	0.95014426	0.982047763	0.960900393
7	0.978558437	0.946681257	0.980451605	0.958183179
8	0.976942515	0.941907128	0.979007186	0.954450582
9	0.975025289	0.935740872	0.977336858	0.949639924
10	0.973383082	0.930028859	0.975943369	0.945187696
11	0.973203571	0.92918728	0.975809749	0.944533109
12	0.972933089	0.927713825	0.975626091	0.943388252
13	0.971770052	0.92174276	0.974805323	0.938749856
14	0.971336609	0.919528336	0.97449868	0.937029983
15	0.970563568	0.918118463	0.973733196	0.935924382
16	0.969814962	0.916752927	0.972991922	0.934853653
17	0.968705844	0.914729061	0.97189371	0.933267045
18	0.96837368	0.914122993	0.97156481	0.932791896
19	0.998381975	0.996525387	0.998460585	0.997256101
20	0.996326889	0.992949488	0.996406406	0.994400126
21	0.995922238	0.992245333	0.996001937	0.993837761
22	0.995556152	0.99160823	0.99563602	0.993328971
23	0.990638643	0.979486717	0.991141574	0.983828081
24	0.98684454	0.972819627	0.987355223	0.978527729
25	0.984950332	0.969489201	0.985465022	0.975880888
26	0.97961584	0.948238919	0.981578431	0.959413141
27	0.978863474	0.945709413	0.980998518	0.957439935
28	0.975024676	0.934400508	0.977812925	0.948608451
29	0.97238115	0.926280855	0.975669575	0.942270535
30	0.971614707	0.922784212	0.975212834	0.939549492
31	0.969329801	0.918628673	0.972950597	0.936288531
32	0.968827187	0.917714522	0.972452972	0.935571196
33	0.96867144	0.917431232	0.972298774	0.935348907

These values are similar to those recorded in the single objective case 3 when only voltage deviation was the targeted objective function suggesting that the multi-objective function considering simultaneously HC and node voltage deviation was ineffective for further reducing the value of the later. The capacity of photovoltaic for this case has also remained similar to those of cases 3 and 4 under specific similar considerations, and as per Table 13 results they still take care of the load demand between 11:00 and 14:00 h. as the grid demand is zero at this period of time,

As per results of Table 14, the voltages at node 18 are also similar to the ones in case 3 when the focus was only on minimizing the node voltage deviation.

### Case 6: Multi-objective considering network losses and V_Dev_

This case study simultaneously minimizes the network losses and voltage deviation in a multi-objective approach and results from Tables 3, 4, 15 and 16 are discussed. From Table 4 results, the network losses with and without the demand response are 1248.63 kWh and 1471.68 kWh respectively. Compared to those from case 1, the network losses in this case have reduced by respectively 11% and 34.76% under similar scenarios. Node voltage deviation minimization has also been achieved for this case at 0.5084 p.u. compared to 0.705 p.u. in case 1 both during DR implementation scenario.

**Table 15:** Capacity of PV with multi-objective considering network losses and V_Dev_.

Without DR				With DR		
Time [h]	PPV1 [MW]	PPV2 [MW]	Grid demand [MWh]	PPVDR1 [MW]	PPVDR2 [MW]	Grid demand DR [MWh]
1	0	0	1.045098	0	0	1.257644
2	0	0	1.010469	0	0	1.215854
3	0	0	1.158346	0	0	1.394373
4	0	0	1.02386	0	0	1.232013
5	0	0	1.22312	0	0	1.472619
6	0.0499	0.056985	1.572828	0.056529	0.0617	1.742678
7	0.059711	0.068189	1.663641	0.067643	0.073831	1.778386
8	0.289081	0.330124	1.849224	0.327483	0.35744	1.699706
9	0.480295	0.548487	1.611112	0.544098	0.59387	1.685806
10	0.901893	1.029943	0.493043	1.021702	1.115163	0.77818
11	0.659093	1.169448	0.057598	0.754158	1.266212	0.244331
12	0.724947	1.347637	0.104626	0.835835	1.459145	0.319836
13	0.705905	1.322064	0.087284	0.813283	1.431456	0.295491
14	0.705482	1.138784	0.113006	0.805266	1.23301	0.312235
15	0.741245	0.846486	0.728139	0.839713	0.916526	1.028913
16	0.333165	0.380468	1.677325	0.377424	0.411949	1.694593
17	0.049725	0.056785	3.307613	0.05633	0.061483	2.585854
18	0.022709	0.025934	3.308229	0.025726	0.02808	2.605307
19	0.013088	0.014947	3.884185	0.014827	0.016183	3.062332
20	0	0	2.98136	0	0	2.364814
21	0	0	3.100546	0	0	2.458407
22	0	0	2.763749	0	0	2.193724
23	0	0	2.335998	0	0	1.879
24	0	0	1.68231	0	0	1.764013

**Table 16:** Node voltage profile for multi-objective optimization considering network losses and V_Dev_.

Without DR			With DR	
Nodes	Mean voltage [p.u]	Minimum voltage [p.u]	Mean voltage DR [p.u]	Minimum voltage DR [p.u]
1	1	1	1	1
2	0.998685579	0.99705349	0.998712615	0.997675089
3	0.992678729	0.983070545	0.992850337	0.986659476
4	0.989919013	0.975669439	0.990195647	0.980851275
5	0.987271701	0.968357391	0.987657603	0.975117046
6	0.980208523	0.95014426	0.980840712	0.960833097
7	0.978558437	0.946681257	0.979241979	0.958115037
8	0.976942515	0.941907128	0.977794479	0.954379879
9	0.975025289	0.935740872	0.976120221	0.949565533
10	0.973383082	0.930028859	0.974723183	0.945109603
11	0.973203571	0.92918728	0.97458908	0.944454337
12	0.972933089	0.927713825	0.974404603	0.943308195
13	0.971770052	0.92174276	0.973580457	0.938664733
14	0.971336609	0.919528336	0.973272572	0.936942991
15	0.970563568	0.918118463	0.972506018	0.935837287
16	0.969814962	0.916752927	0.971763707	0.934766457
17	0.968705844	0.914729061	0.970663958	0.9331797
18	0.96837368	0.914122993	0.970334599	0.932704508
19	0.998381975	0.996525387	0.998409117	0.997253227
20	0.996326889	0.992949488	0.996354827	0.994397244
21	0.995922238	0.992245333	0.995950336	0.993834877
22	0.995556152	0.99160823	0.995584399	0.993326086
23	0.990638643	0.979486717	0.990813586	0.983809789
24	0.98684454	0.972819627	0.987025812	0.978509336
25	0.984950332	0.969489201	0.985134884	0.975862445
26	0.97961584	0.948238919	0.980257321	0.95934029
27	0.978863474	0.945709413	0.979517671	0.957359313
28	0.975024676	0.934400508	0.975734107	0.948498541
29	0.97238115	0.926280855	0.973131512	0.942138379
30	0.971614707	0.922784212	0.972385393	0.939403469
31	0.969329801	0.918628673	0.970115951	0.936141997
32	0.968827187	0.917714522	0.969616744	0.935424549
33	0.96867144	0.917431232	0.969462057	0.935202225

However, the sizes of photovoltaic capacity have reduced by 6.11% and 14.6% under both no-DR and DR implementation scenarios respectively. The above results strongly suggest that hosting capacity increase might be barely achieved when the multi-objective optimization problem formulation does not specifically consider hosting capacity as one of the objective function.

From Table 16 results, the voltages at node 18 are respectively 0.9141 p.u. and 0.9327 p.u. , figures that are the best minimum voltage out of all the cases.

### Case 7: Multi-objective considering simultaneously maximize HC of solar PV, minimize V_Dev_ and minimize network losses

This case study simultaneously considers all the objectives namely the maximization of HC, and the minimization of both network losses and voltage deviation in a multi-objective framework. Discussions will refer to the results from Tables 3, 4, 17 and 18. Solar PV capacities as per Table 4 are 2754.83 kW and 3322.31 kW for no-DR and DR implementation scenarios respectively. It can be noted that this value is the second highest capacity obtained out of all the cases except in case 1 were the case focused on maximizing HC as a single objective.

**Table 17:** PV capacity and grid demand for multi-objective simultaneous consideration of HC, V_Dev_ and network losses.

Without DR				With DR		
Time [h]	PPV1 [MW]	PPV2 [MW]	Grid demand [MWh]	PPVDR1 [MW]	PPVDR2 [MW]	Grid demand DR [MWh]
1	0	0	1.045098	0	0	1.257644
2	0	0	1.010469	0	0	1.215854
3	0	0	1.158346	0	0	1.394373
4	0	0	1.02386	0	0	1.232013
5	0	0	1.22312	0	0	1.472619
6	0.051043	0.061072	1.567385	0.06065	0.074561	1.689848
7	0.061079	0.07308	1.657115	0.072575	0.089221	1.724181
8	0.295703	0.353804	1.817859	0.351356	0.431947	1.621726
9	0.491297	0.587829	1.559883	0.583762	0.71766	1.604263
10	0.922552	1.10382	0.401654	1.096183	1.347615	0.482332
11	0.635602	1.253333	0	0.757931	1.519261	0
12	0.738358	1.444303	0	0.876144	1.75716	0
13	0.702933	1.416896	0	0.850799	1.706136	0
14	0.741869	1.220469	0	0.875949	1.490027	0
15	0.758224	0.907204	0.651958	0.900927	1.107573	0.781746
16	0.340797	0.407759	1.641438	0.404938	0.497818	1.599752
17	0.050864	0.060858	3.301899	0.060437	0.074299	2.567721
18	0.02323	0.027794	3.305616	0.027602	0.033933	2.597005
19	0.013388	0.016019	3.88265	0.015908	0.019557	3.057476
20	0	0	2.98136	0	0	2.364814
21	0	0	3.100546	0	0	2.458407
22	0	0	2.763749	0	0	2.193724
23	0	0	2.335998	0	0	1.867798
24	0	0	1.68231	0	0	1.713184

**Table 18:** Mean and minimum voltage at nodes for the multi-objective optimization considering HC, V_Dev_ and network losses together without and with DR.

Without DR			With DR	
Nodes	Mean voltage [p.u]	Minimum voltage [p.u]	Mean voltage DR [p.u]	Minimum voltage DR [p.u]
1	1	1	1	1
2	0.998685567	0.997053492	0.998764029	0.997677962
3	0.992678653	0.983070555	0.993177313	0.986677717
4	0.98991889	0.975669455	0.99072739	0.980880894
5	0.987271529	0.968357414	0.988403062	0.975158509
6	0.980208249	0.950144301	0.982046814	0.960900411
7	0.978562085	0.946681521	0.980467688	0.958184031
8	0.976961844	0.941908162	0.979091833	0.954454373
9	0.97506722	0.93574303	0.977520263	0.949648
10	0.973447999	0.930032153	0.976227253	0.945200105
11	0.973272835	0.929190786	0.976112648	0.944546329
12	0.973010625	0.927717734	0.975965168	0.943403013
13	0.971880004	0.921748258	0.975286149	0.938770684
14	0.971458569	0.919534419	0.97503205	0.937053048
15	0.97068562	0.918124555	0.974267042	0.935947474
16	0.969937103	0.916759029	0.973526229	0.934876772
17	0.968828117	0.914735177	0.972428698	0.933290203
18	0.968495993	0.914129113	0.972100003	0.932815066
19	0.998381963	0.996525389	0.998460549	0.997256101
20	0.996326877	0.992949489	0.996406378	0.994400127
21	0.995922226	0.992245335	0.996001911	0.993837761
22	0.99555614	0.991608232	0.995635996	0.993328972
23	0.990638567	0.979486727	0.991141334	0.983828083
24	0.986844463	0.972819637	0.987354997	0.97852773
25	0.984950256	0.969489211	0.985464789	0.975880889
26	0.979611081	0.948238742	0.98155787	0.959412315
27	0.978852431	0.945708931	0.980950464	0.957437928
28	0.974990176	0.934398877	0.97766223	0.948602007
29	0.972328642	0.92627835	0.975439824	0.942260717
30	0.971550806	0.922781159	0.974932974	0.939537556
31	0.969265756	0.918625607	0.972670105	0.936276554
32	0.96876311	0.917711453	0.972172343	0.93555921
33	0.968607354	0.917428161	0.972018102	0.935336917

Additionally, as compared to case 1, in this current case the sizes of photovoltaic are reduced by a tiny 1.5% and 2.11% respectively during no-DR and DR scenarios. This suggests that HC of photovoltaic is not that much effected when all the objectives are being considered simultaneously.

From Table 17 results it can be observed that the grid demand is zero between 11:00 and 14:00 h proving that PV capacities are catering for the network full demand for that period of time. The network losses without and with demand response implementation are 1489.94 kWh and 1314.866 kWh respectively. It can be exerted from the results in Tables 3 and 4 that for this case, the network losses have reduced by 31.31% compared to case 1 but remain acceptably higher than the 1197.10 kWh network losses of case 2 in which the single objective was to minimize network losses. From Table 4, it can be established that node voltage has been kept at their minimal values which are 0.6762233 p.u and. 0.498274 p.u. respectively under no-DR and DR implementation scenarios. Table 18 show that the minimum node voltage at 18th node of the distribution system remains at minimum 0.9141 p.u and 0.932815066 p.u respectively for no-DR and DR implantation scenario.

### Sensitivity analysis

In this case, the sensitivity analysis of all the considered objectives of distribution system is investigated with different participation rates of consumers. The Table 19 represents that the hosing capacity of photovoltaic is increases by 31% as the participation level of consumers for demand response is increasing from zero to 30%. It shows that with the optimal utilization of demand response increases the penetration level of photovoltaic in the distribution system. The network losses are also reduced as the demand response penetration level is increases. The reduction in network losses is 14.50% as the controllable demand for demand response is increase to 30%. The voltage deviation is also improved from 0.67622 to 0.431. Table 19 shows that with the successful implementation of demand response impacts on the improvement of optimization objectives of the distribution system. This is because variability is introduced into the grid by high solar power generation levels. However, the availability of sunlight is determined by weather conditions, which might change throughout the day. Another degree of unpredictability may come from demand response initiatives that motivate customers to modify their electricity usage. The successful modification in the load demand profile, combined with power availability increase the optimal utilization of solar generation.

**Table 19:** Sensitivity analysis for HC of PV, network losses and V_Dev_ with different participation rate of demand response.

DR rates (percentage)							
Cases	0%	5%	10%	15%	20%	25%	30%
CapPPV1 [kW]	1254.199	1313	1373	1433	1490.248	1548	1607
CapPPV2 [kW]	1500.631	1583	1665	1747	1832.067	1917	2001
HC [kW]	2754.83	2896	3038	318	3322.315	3465	3608
Loss [kWh]	1489.95	1434	1387	1347	1314.866	1290	1273
V_Dev_ [p.u.]	0.676223	0.626	0.58	0.537	0.498274	0.463	0.431

## Conclusion

The improved crow search optimization approach for distribution system operation and planning with demand response support is presented in this research work. The carefully shifting of controllable demand while maintaining the objectives and constraints of distribution is very crucial. Seven distinct case studies were used to evaluate the effectiveness of the suggested approach while taking technical conflicting in nature objectives into consideration. The suggested model is examined in each case study with and without demand response support to see that demand response plays an important role in increasing the HC of solar PV and hence improving electric distribution system planning and operation optimization models. The comprehensive analysis of the results indicates that, with the aid of demand response, the suggested distribution system planning and operating models optimize the integration of photovoltaic systems by maximizing the hosting capacity while minimizing the network losses and the voltage deviation for the benefits of both utilities and consumers. Modified crow search optimization achieves fast convergence and better robustness with sensitivities leading to superior outcomes.

In the future work, uncertainty of multiple renewable energy sources such as wind and solar generation, node wise electricity demand and electricity tariff will be considered for the multi-objective operational planning problem of distribution system.

## Data Availability

The data sets associated with the current study will be available from the corresponding author on request.

## Sets/indices

$m,n$: Index for system node identification
$t$: Index for time
$N$: Total number of nodes
$\Psi^{N}$: Set of any node
$\Psi^{T}$: Set of time interval
$\Psi^{PV}$: Set of nodes with installed PV resources

## Parameters

$P_{m,t}^{PV,p.u}$: PV per unit power
$P_{m,t}^{load,bDR}$: Active load demand before DR implementation
$Q_{m,t}^{load,bDR}$: Reactive load demand before DR implementation
$V_{m}^{\min }$/$V_{m}^{Max}$: Minimum/Maximum Node voltage magnitude
$V_{setpt}$: Voltage set point
$Y_{mn}$: Magnitude of the admittance matrix
$$\phi_{nm}$$: Angle admittance
$X$: Feeder line reactance
$\omega_{1}$, $\omega_{2}$ and $\omega_{3}$: Weighting coefficients for power losses, voltage deviation and hosting capacity
$\sigma$: Percentage PV power utilization rate
$\kappa_{m,t}^{Max}$: Maximum load demand flexibility potential

## Variables

$P_{m,t}^{PV}$: PV contribution power
$C_{m}^{PV}$: Nodal PV capacity
$$H_{C}$$: Hosting capacity
$P_{m,t}^{load,aDR}$: Active power demand after DR implementation
$Q_{m,t}^{load,aDR}$: Reactive power demand after DR implementation
$P_{m,t}^{GRID}$: Grid active power
$Q_{m,t}^{GRID}$: Grid reactive power at node *m*, time *t*
$P_{L}$: System’s total power loss
$V_{m,t}^{{}} /V_{n,t}$: Instantaneous node voltage magnitudes
$$V_{Dev}$$: Voltage deviation
$\delta_{m,t} /\delta_{n,t}$: Instantaneous node voltage angles
$\kappa_{m,t}$: Change in load demand flexibility
